# Reduced risk of post ERCP pancreatitis in statin users

**DOI:** 10.1186/s12876-020-01264-5

**Published:** 2020-04-23

**Authors:** Yousaf Bashir Hadi, Syeda Fatima Naqvi, Abdelhai Abdelqader, Justin Kupec, John Nasr

**Affiliations:** West Virginia University, Morgantown, WV 26505 USA

**Keywords:** Post ERCP pancreatitis, Pancreatitis, Statin, Endoscopy, Pancreas

## Abstract

**Background:**

One of the most feared complications of endoscopic retrograde cholangiopancreatography (ERCP), with an incidence of 3.5 to 15%, is post ERCP pancreatitis (PEP). Given the role of statins in the reduction of systemic and pancreatic intraluminal inflammation, we hypothesized that the use of statins may lower the risk of PEP.

**Methods:**

A retrospective cohort study of all patients undergoing ERCP at West Virginia University during the years 2016 and 2017 was performed. Possible association of collected variables with PEP was assessed with Univariate tests and multivariable logistic regression analyses.

**Results:**

A total of 1162 ERCPs were included. Mean age was 60.12 years (SD: 17.5). 51.3% of the participants were female. Two hundred and sixty-three participants underwent more than one ERCP during the study period.

Seven hundred and ninety-nine ERCPs (78.8%) were conducted in participants who were not taking a statin medication at the time of ERCP, while 363 participants were on statin medications at the time of ERCP; 118 and 245 participants were taking high dose statins (atorvastatin 40–80 mg or rosuvastatin 20 mg), and low/medium dose statins (all other statin regimens) at the time of the procedure, respectively.

The overall incidence of PEP in the cohort was 7.3%. In the non-statin and statin groups, 9.5 and 3.4% of participants developed PEP, respectively. On univariate analysis, young age, no statin use, history of PEP, and endoscopic sphincterotomy were found to be significantly associated with the development of PEP. In a binary logistic regression model, young age (*P* = 0.033), history of PEP (*P* = 0.0001, OR 2.41, 95% CI: 1.05–5.51) and endoscopic sphincterotomy (*P* = 0.038, OR 2.85, 95% CI: 1.7–4.78) were found to be associated with increased risk of PEP. Statin usage was found to be protective against PEP, (OR 0.35, 95% CI: 0.18–0.69).

**Conclusion:**

Chronic statin usage is protective against post ERCP pancreatitis, and our findings suggest a potential role of these drugs as prophylactic agents. Randomized controlled trials are needed to establish any potential clinical application.

## Background

Endoscopic retrograde cholangiopancreatography (ERCP) is the diagnostic and therapeutic procedure of choice for many biliary and pancreatic pathologies. More than half a million ERCPs are performed annually in the United States [1]. Although its use as a diagnostic modality has decreased over the course of the last decade due to the emergence of some other non-invasive modalities, it remains the primary therapeutic option for choledocholithiasis, cholangitis and other biliary and pancreatic ailments. Post ERCP pancreatitis (PEP) is the most common complication of this procedure and its incidence has been reported to be up to 15% in various studies [2]. Hyperamylasemia and abdominal pain are common after ERCP, and consensus criteria have been developed to diagnose PEP; the criteria by Cotton et al. is the most widely accepted and utilized [3, 4]. Various risk factors for PEP have been identified, and, in recent years, the use of prophylactic stent placement and the use of rectal non-steroidal anti-inflammatory drugs (NSAIDs) has been linked with a decreased rate of PEP. Rectal NSAIDs and prophylactic stent placement (in high risk population) are now recommended by major academic organizations for the prophylaxis of PEP [5, 6].

Statins are commonly used medications that competitively inhibit 3-hydroxy3-methylglutaryl coenzyme A (HMG-CoA) reductase [7]. They are used both in hypercholesterolemia and cardiovascular disease due to their effect of lowering low density lipoprotein [8]. However, a growing body of scientific evidence now shows that some of these beneficial effects are mediated by pleiotropic, immunomodulatory, and anti-inflammatory effects of statins [9]. These effects have led to interest in exploration of their potential use in diseases mediated by chronic and acute inflammation such as multiple sclerosis, and rheumatoid arthritis, among others [10, 11]. Previous data has shown that chronic statin use may be associated with a reduced risk of acute pancreatitis. We hypothesized that the use of statins, due to their pleiotropic and anti-inflammatory effects, may be associated with a reduced risk of PEP.

## Methods

A retrospective cohort study of all patients who underwent ERCP at West Virginia University Hospital during the years 2016 and 2017 was performed. Patients were included if they were adults (ages 18 and older) and if all ERCP related records were available in the electronic patient chart. The study was reviewed and approved by our institutional review board before commencement. Eligible participants were identified through our health system’s electronic medical record database. Patient charts were reviewed by two independent authors (YH and SN), and relevant variables were extracted, including demographic variables, procedure related variables, complications, and medications.

Post ERCP pancreatitis was defined according to the Cotton definition [4]. These criteria require the presence of new or worsening abdominal pain after ERCP and amylase greater than three times upper limit of normal 24 h after the procedure, requiring hospital stay or extension of stay by 2 days. Pancreatitis severity was assessed by the APACHE II score and by Atlanta criteria [12, 13].

Chronic statin use was defined as statin use for a period of 6 months or longer prior to ERCP. Statins were divided into high dose (atorvastatin 40 mg per day or more or rosuvastatin 20 mg or more per day) and low dose groups (all other statin regimens), as defined by the American College of Cardiology/ American Heart Association guidelines [14]. At our institute, wire guided cannulation is attempted when performing biliary ERCP, and pancreatic duct cannulation is attempted with contrast assistance. Rectal NSAIDs were administered at the discretion of the endoscopist for patients that were deemed high risk. During the majority of the study period, pancreatic stents were placed for patients undergoing biliary ERCP in whom the pancreatic duct was accessed inadvertently, or in patients undergoing pancreatic ERCP who were deemed high risk by the endoscopist.

Rosuvastatin and pravastatin are hydrophilic statins. All other statins, including atorvastatin, simvastatin, and fluvastatin, are lipophilic.

Data was extracted from the charts using RedCap software, which is a HIPAA-compliant, cloud-based data extraction tool [15]. Data analysis was conducted using R statistical package [16]. Categorical data was expressed as frequencies and proportions; means and standard deviations were computed for continuous variables. Chi square tests and T tests were conducted for categorical and continuous variables, respectively, to determine associations between the usage of statins and different extracted variables and population characteristics.

Multivariable logistic regression analysis was then performed to assess independent associations of variables with PEP. All variables with *p* values less than 0.15 were entered into the regression analysis. All p values less than 0.05 were considered significant for the purposes of this study.

## Results

A total of 672 patients underwent a total of 1162 ERCPs during the study period and were included in the study. The mean age of participants was 60.12 years (SD: 17.5) and 51.3% of the participants were female. Two hundred and sixty-three participants underwent more than one ERCP during the study period.

Seven hundred and ninety-nine ERCPs (78.8%) were conducted in participants who were not taking a statin medication at the time of ERCP, while 363 procedures were conducted in participants who were on statin medications. Of the 363 procedures, 118 and 245 participants were taking high dose statins and low/medium dose statins at the time of the procedure, respectively. 53% of ERCPs were performed as inpatient. Baseline characteristics of the study population are presented in Table 1.

**Table 1:** Characteristics of the study population

Variable	N (%)
Age	60.12 (SD 17.5)
Female	342 (51.30%)
Inpatient procedure	615 (53.01%)
Statin use	
High dose	118 (10.15%)
Low dose	245 (21.08%)
No statin use	799 (78.8%)
Multiple ERCPs	263 (22.63%)
History of acute pancreatitis	357 (30.72%)
History of Post ERCP pancreatitis	72 (6.2%)
Native papilla	790 (68%)

**Fig. 1:**
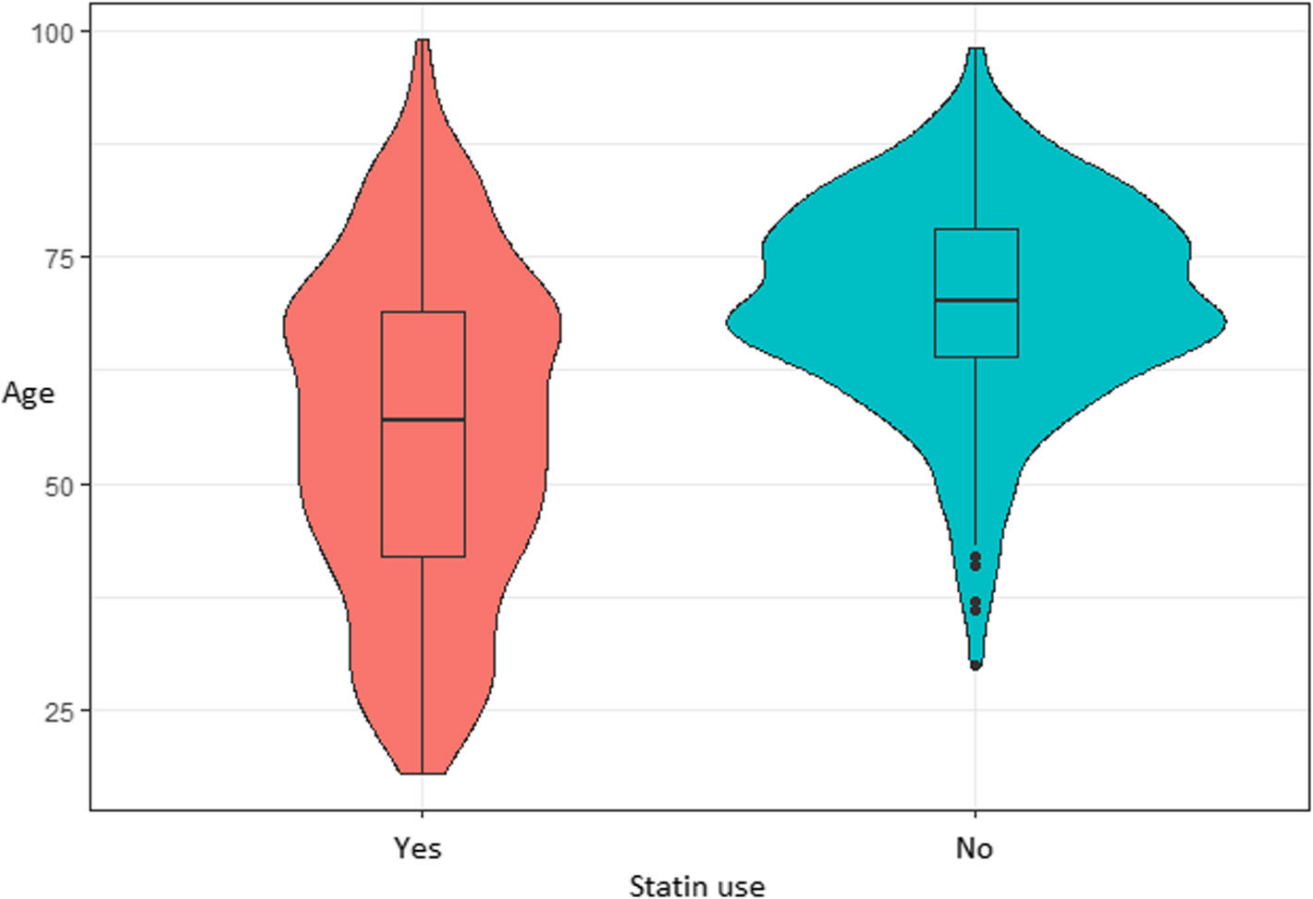
Age distribution violin and box graph of statin users and non-users

The mean age was greater in the statin user group (*p* = < 0.05). Figure 1 delineates the age distribution in the two groups. Gender, common bile duct (CBD) stent placement, endoscopic sphincterotomy, pancreatic duct (PD) stent placement, rectal indomethacin, PD or CBD cannulation, prior history of post ERCP pancreatitis, and history of acute pancreatitis were not statistically different in the statin and non-statin groups (all *p* values > 0.05). Baseline characteristics for statin users and statin non-users are presented in Table 2.

**Table 2:** Baseline characteristics of the population for the ERCP episodes

Variable	Statin group	Non-statin group	*P* value
Age	67.21 (SD: 12.67)	55.81 (SD: 17.99)	< 0.05
Male gender	179 (49.31%)	387 (48.44%)	0.83
PD stent placement	62 (17.08%)	115 (14.39%)	0.27
Rectal Indomethacin use	40 (11.02%)	103 (12.89%)	0.42
History of post ERCP pancreatitis	19 (5.23%)	53 (6.63%)	0.43
PD cannulation	87 (23.97%)	184 (23.03%)	0.78
Inpatient procedures	204 (56.20%)	411 (51.44%)	0.55
Native papilla	240 (66.12%)	550 (68.84%)	0.36

The overall incidence of PEP in the cohort was 7.3%. PEP in the non-statin and statin groups, respectively, was 9.5 and 3.4%. On univariate analysis, young age, no statin use, endoscopic sphincterotomy, and history of post ERCP pancreatitis were found to be significantly associated with the development of PEP (all *p* values < 0.05). Univariate analyses are detailed in Tables 3 and 4. A logistic regression model was fitted, that incorporated all variables with p values less than 0.15 on univariable analyses. In the logistic regression model, young age (*P* = 0.033), history of PEP (*P* = 0.0001, OR 2.41, 95% CI: 1.05–5.51) and endoscopic sphincterotomy (*P* = 0.038, OR 2.85, 95% CI: 1.7–4.78) were found to be associated with increased risk of PEP. Statin usage was found to be protective against PEP (OR 0.35, 95% CI: 0.18–0.69) Table 4.

**Table 3:** Univariate analyses of the association of variables with PEP

Variable	Univariate analysis (*p* value)
Age	0.00078
Gender	0.379
Statin use	0.00025
Sphincterotomy	0.000385
PD stent placement	0.1535
Rectal Indomethacin use	0.4842
History of post ERCP pancreatitis	0.297
History of acute pancreatitis	0.79
PD cannulation	0.4758
Inpatient vs outpatient procedure	0.17
Native papilla	0.208

**Table 4:** Odds Ratios and Confidence intervals for the variables included in the multivariable logistic regression model

Variable	Odds Ratio	95% Confidence Interval	*p* value
Age	0.99	(0.97–0.99)	0.033
Statin use	0.35	(0.18–0.69)	0.002
Sphincterotomy	2.85	(1.7–4.78)	0.0001
History of post ERCP pancreatitis	2.41	(1.051–5.507)	0.038
Statin Use by intensity^a^			
No statin use	Reference	Reference	Reference
Low intensity statin	0.43	0.208–0.898	0.025
High intensity statin	0.20	0.047–0.820	0.026
Statin Use by type^a^			
No statin use	Reference	Reference	Reference
Lipophilic statin use	0.4	0.193–0.830	0.014
Hydrophilic statin use	0.23	0.056–0.982	0.047

^a^Separate logistic regression model incorporating this variable

Previously reported factors associated with PEP (pancreatic cannulation, native papilla, and rectal indomethacin use) were then used as covariates in the regression model above. No changes in the independent association from the above model were noted.

A subgroup analyses revealed that there was no difference between the incidence of PEP between the hydrophilic and lipophilic groups (*p* = 0.53). A separate multivariable logistic regression model categorizing statin therapy into high dose and low dose, with no statin use as reference, showed a trend toward a more protective effect with high dose statins relative to low dose statins. However a subgroup analyses of only patients who were on statin therapy did not show a statistical difference between the two groups on univariable analyses (*p* = 0.3).

No patient in our study developed severe post ERCP pancreatitis.

## Discussion

We have found an independent association of chronic statin use with a decreased incidence of post ERCP pancreatitis. This is one of the first reports of such a protective effect of statin use on the development of post ERCP pancreatitis. This association was robust and stayed significant after controlling for possible confounders.

Some data has shown that chronic statin use may be associated with a reduced risk of acute pancreatitis [17]. Similarly, a small study noted that statin use was associated with a positive effect on computed tomography severity index of patients with acute pancreatitis without any change in clinical outcomes [18]. A recent study has also noted a protective effect of statins in endoscopic ultrasound guided fine needle procedures of the pancreas [19]. Similar mechanisms including inflammation and acidosis in the pancreatic microcirculation lay at the heart of both acute pancreatitis and PEP. As we now understand that the anti-inflammatory effects of statins are directly linked to their therapeutic effects, they should hold at least some theoretical promise in decreasing the incidence of PEP.

Some reports have suggested an association of statins and reduced PEP [20, 21], however, they did not control for confounders including rectal NSAID use, PD cannulation, and PD stent placement, as well as known risk factors for the development of PEP including a prior history of PEP, and cannulation of pancreas. Mahmid et al. reported an odds ratio of development of PEP in the statin group of 0.318 [95% CI 0.169–0.597], *p* < 0.01, compared to non-users. In the other conference abstract by Martinez-Moneo et al., an odds ratio of 3.56 was reported in statin non-users for development of PEP [95% CI 1.19–10.68], p-0.023). A recent report by Hakuta et al. has shown conflicting results as they did not find an association between statin usage and risk of PEP in their retrospective cohort [22]. In our study, after controlling for all possible confounders, a high protective effect of use of statins on development of PEP was observed. Therefore, it appears that the potential role of statins as prophylactic agents remains controversial. One reason for the variability in findings of studies may be racial differences, as all other studies, apart from Hakuta et al., have been conducted on western, primarily Caucasian populations. Previous studies have noted racial differences in effects of statins [23]. Prospective randomized trials are needed to explore and affirm any potential role of statins for prophylaxis of PEP.

In our multivariate logistic regression analysis, young age, history of post ERCP pancreatitis, and sphincterotomy were independently associated with increased risk of post ERCP pancreatitis, consistent with previous data. In our model, rectal indomethacin use had a trend towards a protective effect but did not reach statistical significance. This finding is likely explained by our institutional practice during the study period; we administered rectal indomethacin only to patients that were deemed high risk by the endoscopist. Although some previous data was heterogenous regarding the protective effect of rectal NSAIDs, robust data is now available cementing their protective effect and prophylactic role. Our study finds that the protective role of statins is unaffected by pancreatic stent placement and rectal NSAID administration, hinting that any possible prophylactic role of statin administration may in fact be additive or adjunctive to these interventions.

This risk reduction of PEP in statin users may be due to the immunomodulatory and pleiotropic effects of statins discussed above. We hypothesize that such an anti-inflammatory effect may be responsible for this protective effect of statins in PEP; as PEP has been shown to be orchestrated by inflammatory disturbances in the pancreatic microcirculation.

The strengths of our study include control of possible confounders, and the study of both high and low doses of statins. Our study is limited by its retrospective cohort design, and we were thus unable to evaluate the effect of a single dose prophylactic statin intake on the incidence of PEP. Patients included in the study self-reported their statin therapy when they completed their assessment on the day of their outpatient procedure. For inpatients, statin administration was documented in the charts within 24 h. Statins cause both acute and chronic changes in metabolism, inflammatory state, and vascular morphology. Studies have shown that many effects of statins last more than 2 days after discontinuation, however, some acute effects of statins may be mitigated by missing a dose [24]. This may introduce some bias, however, any effect introduced by ‘wearing away’ of some therapeutic effect of statins should drive the results towards null hypothesis, and therefore it should not affect the conclusions derived from this study.

## Conclusions

In conclusion, our study finds a protective effect of chronic statin use on the incidence of PEP. Our study points towards a potential role of statins as pharmacologic prophylaxis agents in patients undergoing ERCP. Previous studies in cardiovascular disease have shown that statin drugs produce at least some of their pharmacological effects acutely, hence they may be able to be exploited in PEP prophylaxis. Further prospective randomized studies are needed to determine any such role of statins as prophylactic agents.

## Data Availability

The datasets used and/or analysed during the current study are available from the corresponding author on reasonable request.

## Abbreviations

ERCP: Endoscopic retrograde cholangiopancreatography
PEP: Post ERCP pancreatitis
NSAIDS: Non-steroidal anti-inflammatory drugs
